# Supplementary material for: Comprehensive genomic analysis reveals virulence factors and antibiotic resistance genes in Pantoea agglomerans KM1, a potential opportunistic pathogen
Source: PLoS One. 2021 Jan 6;16(1):e0239792. doi: 10.1371/journal.pone.0239792 (PMC7787473; doi:10.1371/journal.pone.0239792)
Supplement: S1 Fig — Colors refer to the percentage of the complete single-copy orthologs (light blue), complete duplicated orthologs (blue), fragmented or incomplete orthologs (yellow), and missing orthologs (red). (DOCX) [file pone.0239792.s001.docx]

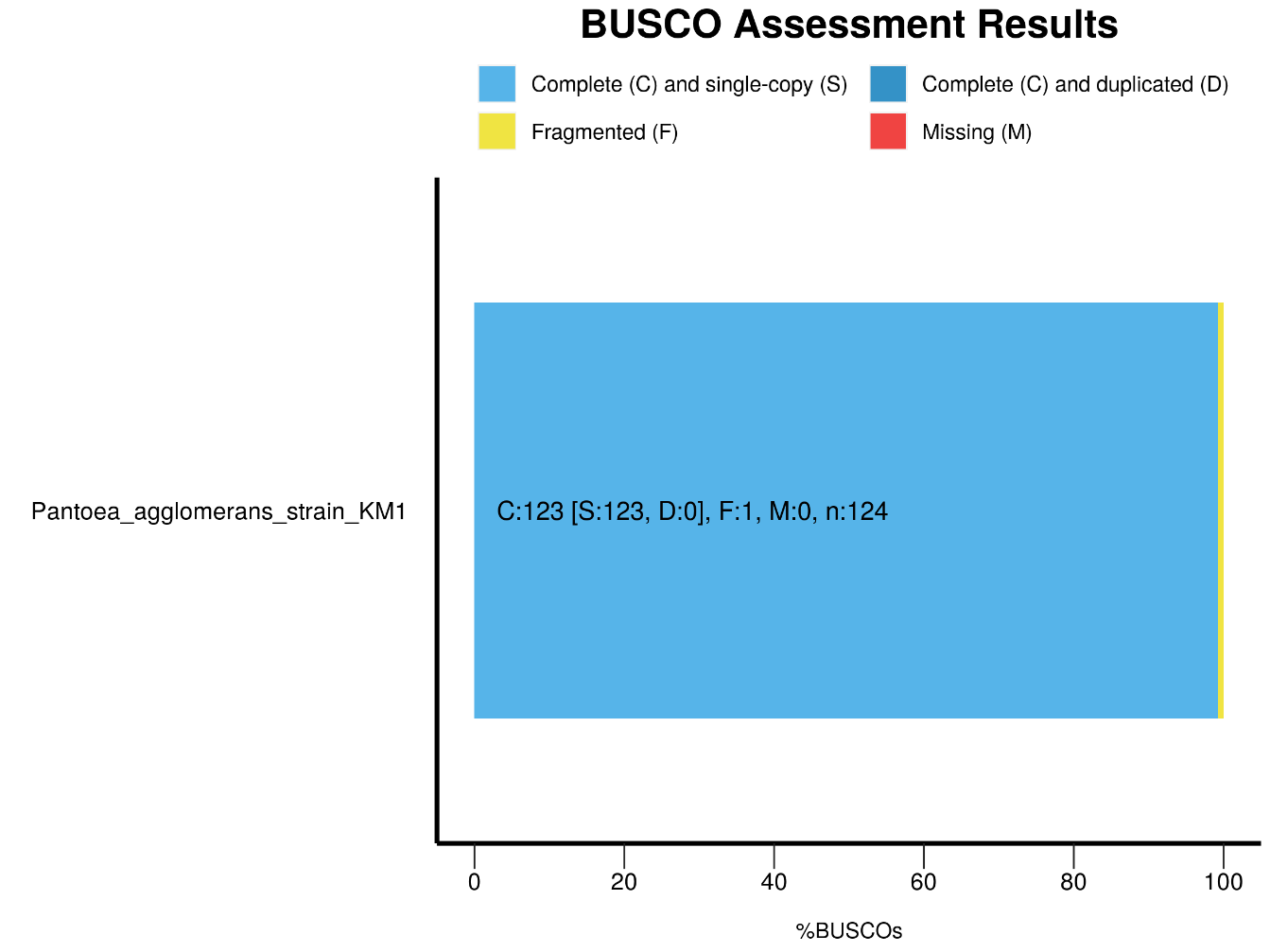


*Pantoea agglomerans* KM1

**S1 Fig. Assessment of the completeness of genome assembly and annotation of *P. agglomerans* strain KM1 using BUSCO.** Colors refer to the percentage of the complete single-copy orthologs (light blue), complete duplicated orthologs (blue), fragmented or incomplete orthologs (yellow), and missing orthologs (red).
