# Supplementary material for: Comprehensive genomic analysis reveals virulence factors and antibiotic resistance genes in Pantoea agglomerans KM1, a potential opportunistic pathogen
Source: PLoS One. 2021 Jan 6;16(1):e0239792. doi: 10.1371/journal.pone.0239792 (PMC7787473; doi:10.1371/journal.pone.0239792)
Supplement: S2 Fig — Bar chart shows the percentage of subsystem coverage with green bar corresponding to the percentage of proteins involved. The pie chart shows the distribution and count of each SEED subsystem feature. (DOCX) [file pone.0239792.s002.docx]

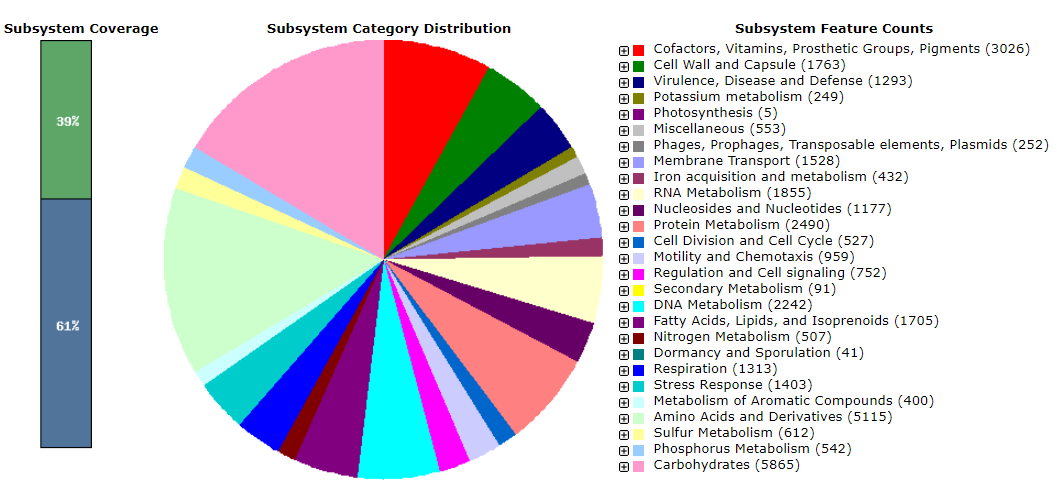


**S2 Fig. Subsystem category distribution of *P*. *agglomerans* KM1 draft genome based on the SEED database.** Bar chart shows the percentage of subsystem coverage with green bar corresponding to the percentage of proteins involved. The pie chart shows the distribution and count of each SEED subsystem feature.
