# Supplementary material for: Comprehensive genomic analysis reveals virulence factors and antibiotic resistance genes in Pantoea agglomerans KM1, a potential opportunistic pathogen
Source: PLoS One. 2021 Jan 6;16(1):e0239792. doi: 10.1371/journal.pone.0239792 (PMC7787473; doi:10.1371/journal.pone.0239792)
Supplement: S3 Fig — (A) Heap’s law chart representation regarding conserved genes and total genes in P. agglomerans genomes. (B) Diagram of new genes vs. unique genes in relation to number of genomes embedded in the analysis. (DOCX) [file pone.0239792.s003.docx]

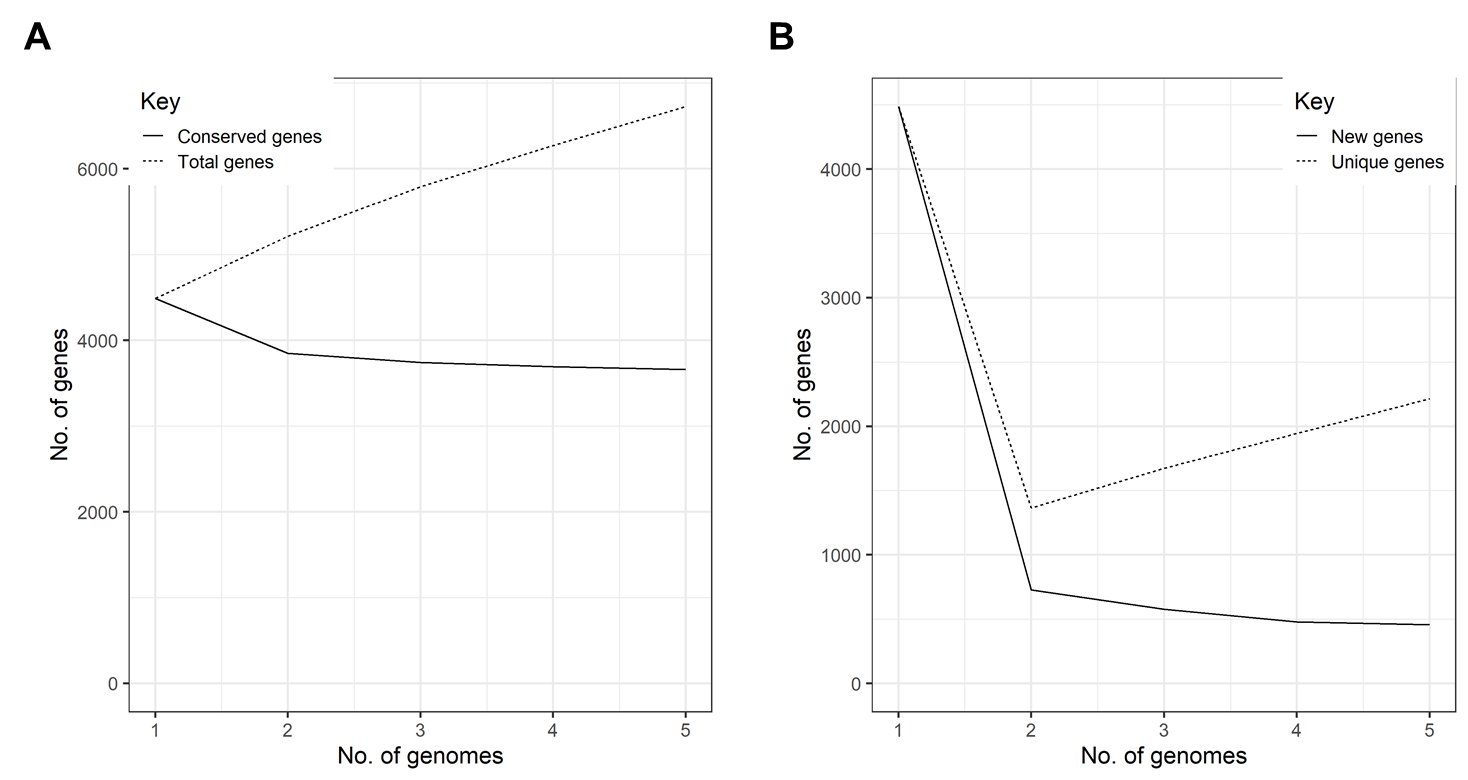


**S3 Fig. Core and pan-genome analysis of five *P*. *agglomerans* strains.** (A) Heap’s law chart representation regarding conserved genes and total genes in *P*. *agglomerans* genomes. (B) Diagram of new genes vs. unique genes in relation to number of genomes embedded in the analysis.
