# Supplementary material for: Comprehensive genomic analysis reveals virulence factors and antibiotic resistance genes in Pantoea agglomerans KM1, a potential opportunistic pathogen
Source: PLoS One. 2021 Jan 6;16(1):e0239792. doi: 10.1371/journal.pone.0239792 (PMC7787473; doi:10.1371/journal.pone.0239792)
Supplement: S4 Fig — Different functional groups of coding sequences of phage origins are denoted by different colors, according to their function. (DOCX) [file pone.0239792.s004.docx]

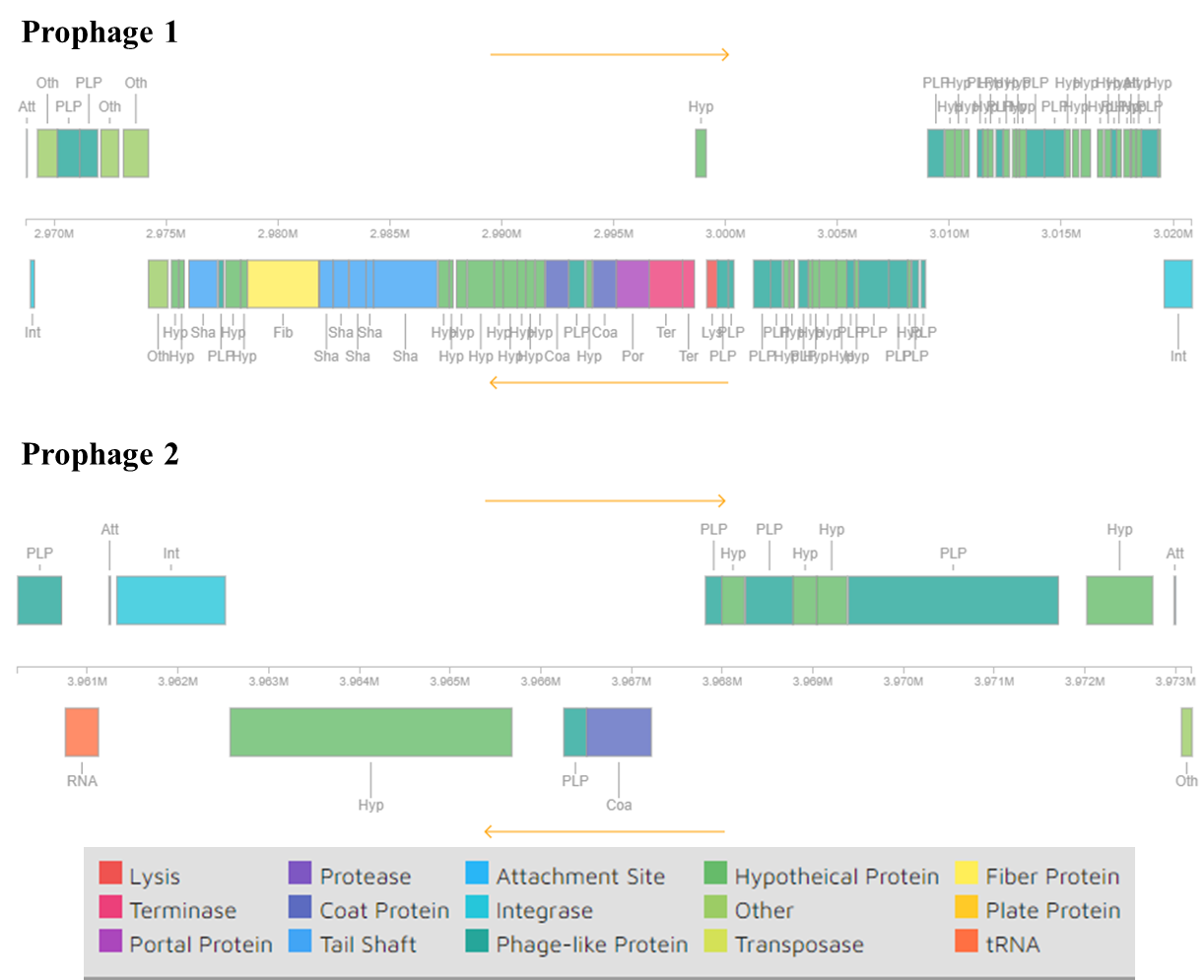


**S4 Fig. Linear genomic map of *P*. *agglomerans* KM1 phage-associated regions obtained with PHASTER.** Different functional groups of coding sequences of phage origins are denoted by different colors, according to their function.
