# Supplementary material for: Comprehensive genomic analysis reveals virulence factors and antibiotic resistance genes in Pantoea agglomerans KM1, a potential opportunistic pathogen
Source: PLoS One. 2021 Jan 6;16(1):e0239792. doi: 10.1371/journal.pone.0239792 (PMC7787473; doi:10.1371/journal.pone.0239792)
Supplement: S5 Fig — Lane 1: 100 bp DNA marker, Lane 2: Hcp negative control, Lane 3: Hcp in KM1, Lane 4: VgrG negative control, Lane 5: VgrG in KM1. The amplicon size of T6SS effectors were 1301 bp (Hcp) and 1011 bp (VgrG). (DOCX) [file pone.0239792.s005.docx]

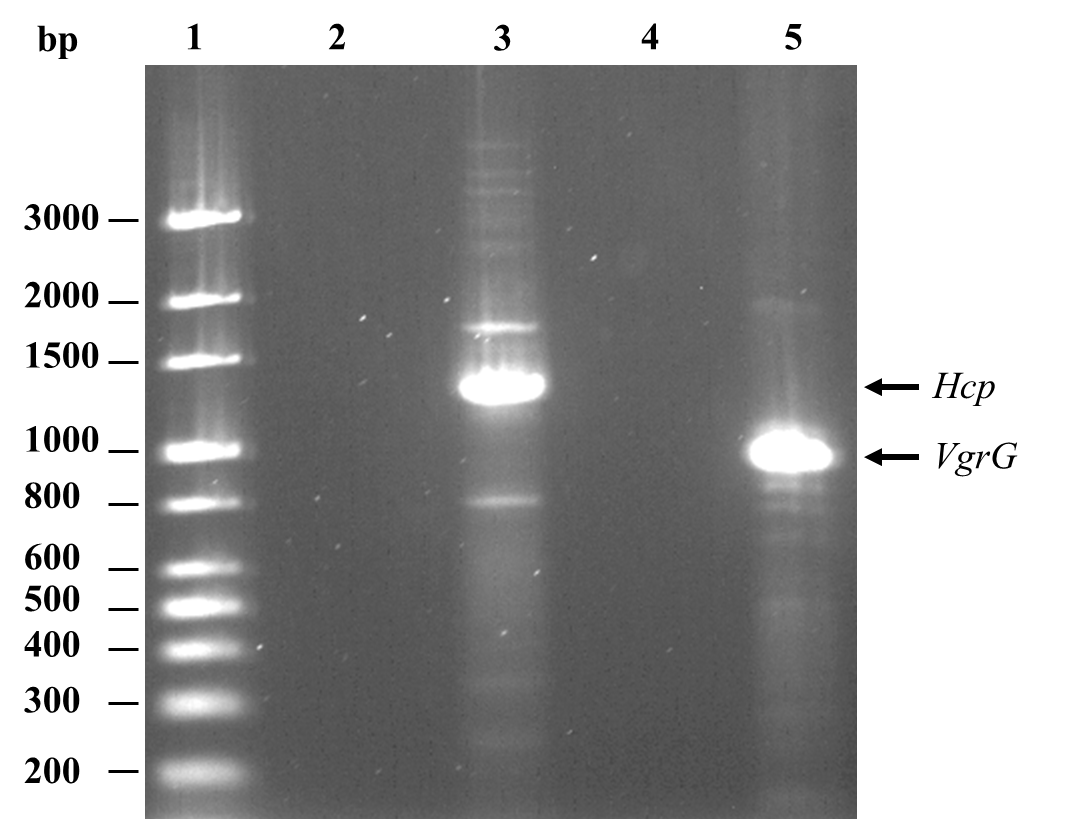


**S5 Fig. Gel electrophoresis of amplified PCR products of type VI secretion system in *P*. *agglomerans* KM1.**  Lane 1: 100 bp DNA marker, Lane 2: *Hcp* negative control, Lane 3: *Hcp* in KM1, Lane 4: *VgrG* negative control, Lane 5: *VgrG* in KM1. The amplicon size of T6SS effectors were 1301 bp (*Hcp)* and 1011 bp (*VgrG*).
