# Supplementary material for: Comprehensive genomic analysis reveals virulence factors and antibiotic resistance genes in Pantoea agglomerans KM1, a potential opportunistic pathogen
Source: PLoS One. 2021 Jan 6;16(1):e0239792. doi: 10.1371/journal.pone.0239792 (PMC7787473; doi:10.1371/journal.pone.0239792)
Supplement: S3 Table — (DOCX) [file pone.0239792.s008.docx]

| Test | Results |
| --- | --- |
| Source information | |
| Kimchi type | cabbage (Baechu) |
| Kimchi pH | 4.5 |
| Ingredients | cabbage, red pepper powder, garlic, ginger, and fermented fish sauce |
| Phenotypic characterization | |
| Colony morphology | Yellow glistening rod-shaped |
| Gram-staining | Negative |
| Size | 2 (±) μm |
| Growth media | Luria-Bertani^1^, de Man-Rogosa-Sharpe^1^, Mueller-Hinton^1^, MacConkey^2^, 5% Blood agar^2^ |
| Growth conditions | 37°C/4°C, facultative aerobic (LB a/b), and 5% CO2 (37°C) |
| Growth rate | 33 min. (37°C, LB) |
| Biochemical characterization | |
| Oxidase | - |
| Catalase | + |
| β-galactosidase (ONPG) | + |
| Arginine dihydrolase (ADH) | - |
| Lysine decarboxylase (LDC) | - |
| Ornithine decarboxylase (ODC) | - |
| Citrate utilization (CIT) | + |
| H_2_S production (H2S) | - |
| Urease (URE) | - |
| Tryptophan deaminase (TDA) | - |
| Indole production (IND) | - |
| Acetoin production (VP) | + |
| Gelatinase (GEL) | + |
| D-glucose (GLU) | + |
| D-mannitol (MAN) | + |
| Inositol (INO) | - |
| D-sorbitol (SOR) | - |
| L-rhamnose (RHA) | + |
| D-sucrose (SAC) | + |
| D-melibiose (MEL) | - |
| Amygdalin (AMY) | + |
| L-arabinose (ARA) | + |
| Lactose^3^ | + |
| Identification | |
| % identity (API 20E) | *Pantoea* spp. group 3, 93.8% |
| % identity (16S rRNA) | *Pantoea* *agglomerans*, 100% |

**S3 Table. Biochemical identification of *P*. *agglomerans* KM1 using API 20E test kit.**

^1^ Cultured in both agar and broth; ^2^ Cultured in agar only; ^3^ Determined on MacConkey agar
