# Supplementary material for: Comprehensive genomic analysis reveals virulence factors and antibiotic resistance genes in Pantoea agglomerans KM1, a potential opportunistic pathogen
Source: PLoS One. 2021 Jan 6;16(1):e0239792. doi: 10.1371/journal.pone.0239792 (PMC7787473; doi:10.1371/journal.pone.0239792)
Supplement: S4 Table — (DOCX) [file pone.0239792.s009.docx]

**S4 Table.** **Genotypic antibiotic resistance gene profile of *P.* *agglomerans* KM1.**

| **Antibiotic Resistance Ontology** | **Gene symbol** | **Gene name** | **AMR gene family** | **Drug class** | **Resistance mechanism** | **% identity** | **Location** | **Locus tag** |
| --- | --- | --- | --- | --- | --- | --- | --- | --- |
| ARO:3000518 | *CRP* | cAMP-activated global transcriptional regulator CRP | resistance-nodulation-cell division (RND) antibiotic efflux pump | penam, macrolide antibiotic, fluoroquinolone antibiotic | antibiotic efflux | 85 | Chromosome | HBB05_RS02420 |
| ARO:3003950 | *msbA* | Lipid A export ATP-binding/permease protein MsbA | ATP-binding cassette (ABC) antibiotic efflux pump | nitroimidazole antibiotic | antibiotic efflux | 92 | Chromosome | HBB05_RS04720, HBB05_RS10365 |
| ARO:3003923 | *oqxB* | multidrug efflux RND transporter permease subunit | resistance-nodulation-cell division (RND) antibiotic efflux pump | tetracycline, nitrofuran, glycylcycline, fluoroquinolone, diaminopyrimidine antibiotic | antibiotic efflux | 82 | Chromosome | HBB05_RS12805 |
| ARO:3000074 | *emrB* | multidrug efflux MFS transporter permease subunit | major facilitator superfamily (MFS) antibiotic efflux pump | fluoroquinolone antibiotic | antibiotic efflux | 80 | Chromosome | HBB05_RS18530 |
| ARO:3000027 | *emrA* | multidrug efflux MFS transporter permease subunit | major facilitator superfamily (MFS) antibiotic efflux pump | fluoroquinolone antibiotic | antibiotic efflux | 80 | Chromosome | HBB05_RS18525 |
| ARO:3002986 | *bacA* | undecaprenyl-diphosphate phosphatase | undecaprenyl pyrophosphate related proteins | peptide antibiotic | antibiotic target alteration | 79 | Chromosome | HBB05_RS01340 |
| ARO:3000792 | *mdtA* | MdtA/MuxA family multidrug efflux RND transporter periplasmic adaptor subunit | resistance-nodulation-cell division (RND) antibiotic efflux pump | aminocoumarin antibiotic | antibiotic efflux | 77 | Chromosome | HBB05_RS16480 |
| ARO:3000793 | *mdtB* | MdtB/MuxB family multidrug efflux RND transporter permease subunit | resistance-nodulation-cell division (RND) antibiotic efflux pump | aminocoumarin antibiotic | antibiotic efflux | 77 | Chromosome | HBB05_RS16485 |
| ARO:3000793 | *mdtC* | multidrug efflux RND transporter permease subunit | resistance-nodulation-cell division (RND) antibiotic efflux pump | aminocoumarin antibiotic | antibiotic efflux | 77 | Chromosome | HBB05_RS16490 |
| ARO:3000702 | *acrR* | multidrug efflux transporter transcriptional repressor | resistance-nodulation-cell division (RND) antibiotic efflux pump | tetracycline, rifamycin, phenicol, glycylcycline, cephalosporin, penam, triclosan, fluoroquinolone antibiotic | antibiotic efflux, antibiotic target alteration | 75 | Chromosome | HBB05_RS08680 |
| ARO:3000491 | *acrD* | multidrug efflux RND transporter permease | resistance-nodulation-cell division (RND) antibiotic efflux pump | aminoglycoside antibiotic | antibiotic efflux | 75 | Chromosome | HBB05_RS17775 |
| ARO:3004074 | *MuxB* | MdtB/MuxB family multidrug efflux RND transporter permease subunit | resistance-nodulation-cell division (RND) antibiotic efflux pump | macrolide antibiotic, tetracycline antibiotic, monobactam, aminocoumarin antibiotic | antibiotic efflux | 75 | Chromosome | HBB05_RS16485 |
| ARO:3002985 | *arnA* | Bifunctional polymyxin resistance protein | pmr phosphoethanolamine transferase | peptide antibiotic | antibiotic target alteration | 76 | Plasmid pKM1_3 | HBB05_RS22975 |
